# The receptor protein tyrosine phosphatase CLR-1 is required for synaptic partner recognition

**DOI:** 10.1371/journal.pgen.1007312

**Published:** 2018-05-09

**Authors:** Aruna Varshney, Kelli Benedetti, Katherine Watters, Raakhee Shankar, David Tatarakis, Doris Coto Villa, Khristina Magallanes, Venia Agenor, William Wung, Fatima Farah, Nebat Ali, Nghi Le, Jacqueline Pyle, Amber Farooqi, Zanett Kieu, Martina Bremer, Miri VanHoven

**Affiliations:** 1 Department of Biological Sciences, San Jose State University, San Jose, CA, United States of America; 2 Department of Mathematics and Statistics, San Jose State University, San Jose, CA, United States of America; University of California San Francisco, UNITED STATES

## Abstract

During neural circuit formation, most axons are guided to complex environments, coming into contact with multiple potential synaptic partners. However, it is critical that they recognize specific neurons with which to form synapses. Here, we utilize the split GFP-based marker Neuroligin-1 GFP Reconstitution Across Synaptic Partners (NLG-1 GRASP) to visualize specific synapses in live animals, and a circuit-specific behavioral assay to probe circuit function. We demonstrate that the receptor protein tyrosine phosphatase (RPTP) *clr-1* is necessary for synaptic partner recognition (SPR) between the PHB sensory neurons and the AVA interneurons in *C*. *elegans*. Mutations in *clr-1/RPTP* result in reduced NLG-1 GRASP fluorescence and impaired behavioral output of the PHB circuit. Temperature-shift experiments demonstrate that *clr-1/RPTP* acts early in development, consistent with a role in SPR. Expression and cell-specific rescue experiments indicate that *clr-1/RPTP* functions in postsynaptic AVA neurons, and overexpression of *clr-1/RPTP* in AVA neurons is sufficient to direct additional PHB-AVA synaptogenesis. Genetic analysis reveals that *clr-1/RPTP* acts in the same pathway as the *unc-6/Netrin* ligand and the *unc-40/DCC* receptor, which act in AVA and PHB neurons, respectively. This study defines a new mechanism by which SPR is governed, and demonstrates that these three conserved families of molecules, with roles in neurological disorders and cancer, can act together to regulate communication between cells.

## Author summary

The nervous system is required for many body functions including perception, behavior and thought. Cells in the nervous system called neurons function in interconnected groups called circuits to carry out these basic functions. While we have learned a great deal about how circuits function, we know much less about how they are set up during development. Discovering the mechanisms that organize these neural circuits could help us to understand neurological disorders that may result from defects in this process. Our work has identified a key role for the cell surface molecule CLR-1 in a critical step in the formation of neural circuits: the recognition between neurons that must link together. We find that CLR-1 acts with the ligand Netrin and its receptor Deleted in Colorectal Cancer (DCC) to mediate communication between adjacent cells. Interestingly, all three of these molecules have been linked to schizophrenia and to cancer, indicating that our discovery may help inform our understanding of these diseases.

## Introduction

Perception, thought, and behavior all rely on the faithful transfer of information between neurons. During development, neurons form circuits through a series of well-characterized steps, including neuronal migration, guidance of axons and dendrites into target regions, and finally the formation of synapses between presumptive neuronal partners. However, within a target region, most neurites contact many potential partners. To form functional circuits, neurons must faithfully recognize and form synapses only with the correct neuronal partners [1, 2]. Relatively little is understood about this process of synaptic partner recognition (SPR), and many of the molecular mechanisms involved remain unknown.

**Fig 1:**
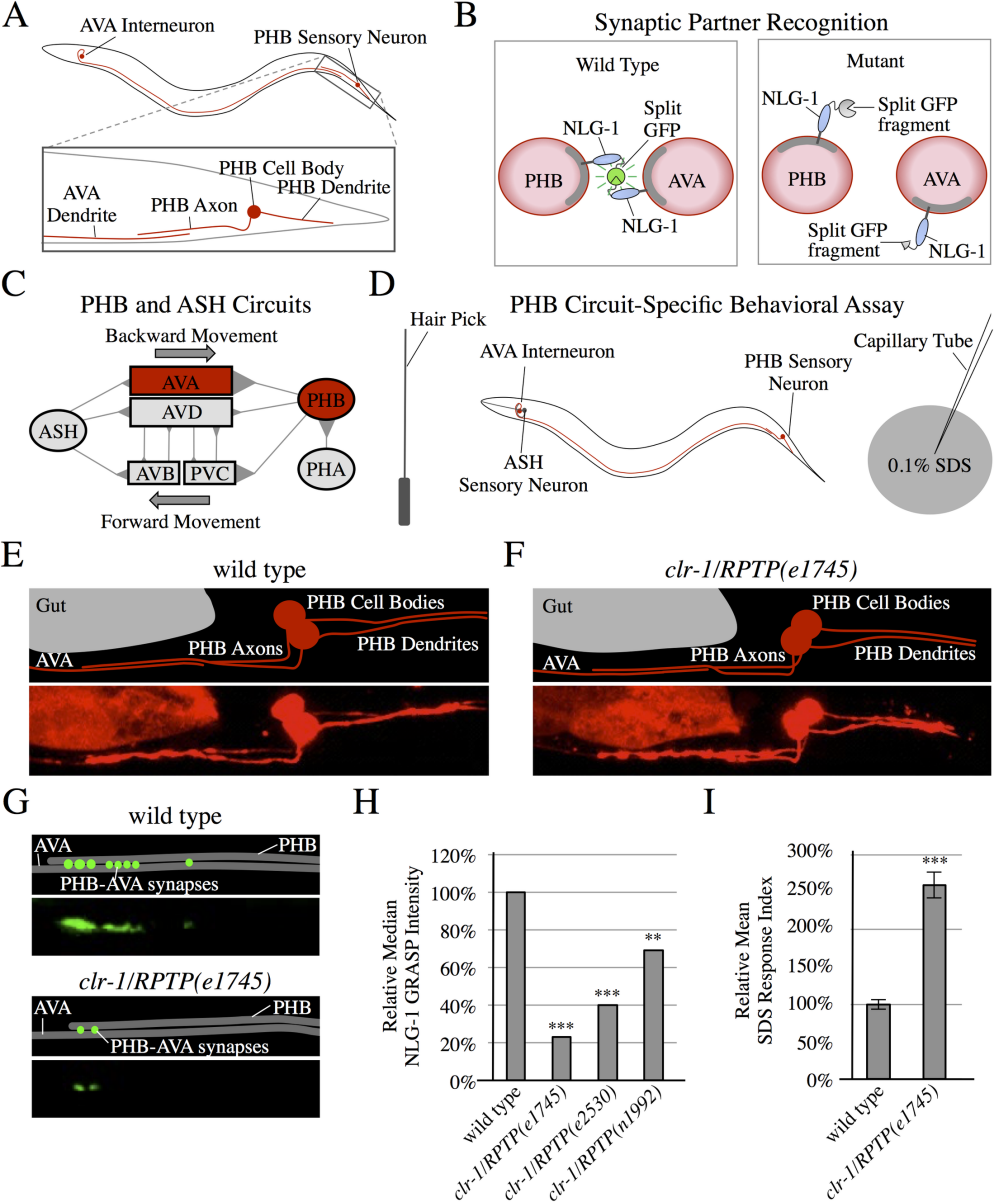
*clr-1/RPTP* mutants display defective synaptic partner recognition. (A) Schematic diagrams of PHB and AVA neurons. (B) Schematic diagrams of the trans-synaptic marker NLG-1 GRASP in pre- and postsynaptic neurites (red circles represent cross-sections of neurites in a region with *en passant* synapses). Split GFPs are linked to the synaptically localized protein NLG-1, so that specific synapses are labeled with green fluorescence in wild-type animals. If a neurite fails to form synapses with the correct partner, NLG-1 GRASP will not reconstitute. (C) Diagram of the PHB and ASH chemosensory circuits including synapses (triangles) connecting sensory neurons (ovals) and interneurons (rectangles) [4, 10, 52]. (D) Outline of a behavioral assay that tests PHB circuit function. Backward movement is induced with a nose touch. Function of PHB-AVA synapses halts backward movement in response to 0.1% SDS. (E and F) Schematics and micrographs of mCherry-labeled PHB neurons and AVA neurites in wild-type (E) and *clr-1/RPTP(e1745)* mutant animals (F), displaying normal morphology and axon guidance to the ventral nerve cord, followed by anterior projection. (G) Schematics and micrographs of PHB-AVA NLG-1 GRASP in wild-type and *clr-1/RPTP(e1745)* mutant animals, showing reduced synapses in *clr-1/RPTP(e1745)* mutant animals. (H) Quantification of NLG-1 GRASP fluorescence demonstrates a reduction in *clr-1/RPTP* mutant animals including *clr-1/RPTP(e1745)*, *clr-1/RPTP(e2530)*, and *clr-1/RPTP(n1992)* as compared with wild type (n>80 except for the low-brood size allele *e2530*, (n = 38)). ***P<0.001, **P<0.01, Mann-Whitney U-test, comparison to wild-type. *P*-values were adjusted for multiple comparisons using the Hochberg method. 95% confidence intervals for the medians are included in S1 Table. (I) *clr-1/RPTP(e1745)* mutants display a defect in the behavioral response to SDS (n>75). ***P<0.001, t-test, comparison to wild-type. Error bars are standard error of the mean (SEM).

To discover molecular pathways that mediate SPR, we focus on the phasmid sensory circuit in *Caenorhabditis elegans* hermaphrodites, which mediates avoidance of toxin-producing *Streptomyces* bacteria [3] and other repulsive cues [4, 5]. Specifically, we study synapses between the PHB sensory neurons and AVA interneurons in *C*. *elegans* (Fig 1A). The left and right PHBs extend axons through a neurite bundle containing approximately 30 potential partners, yet selectively form the majority of their synapses with AVA and PVC interneurons, which control backward and forward movement [6–9]. Here, we study PHB-AVA synapses utilizing the split GFP-based trans-synaptic marker NLG-1 GRASP to visualize specific sensory synapses (Fig 1B) [10, 11], and a circuit-specific behavioral assay to probe circuit function (Fig 1C and 1D) [4, 10]. The stably expressed NLG-1 GRASP marker specifically labels PHB-AVA synapses in live animals without affecting the behavioral output of the circuit [10].

We previously determined that UNC-6/Netrin acts as a retrograde juxtacrine signal from presumptive postsynaptic AVA interneurons to presumptive presynaptic PHB neurons. PHB neurons receive this signal via the UNC-40/DCC receptor, specifying PHBs as AVAs’ synaptic partners [10]. However, it was not known if other molecules were required for SPR, and the mechanism by which AVA neurons receive the SPR signal remained unknown.

Here, we show that the *clr-1* gene plays a crucial role in SPR. *clr-1* encodes a receptor protein tyrosine phosphatase (RPTP) with extracellular domains similar to those in the Leukocyte common Antigen-Related protein (LAR) family of RPTPs [12]. We demonstrate that *clr-1/RPTP* is necessary for formation of PHB-AVA synapses, and overexpression promotes increased synapse formation between the two neurons. CLR-1/RPTP acts in postsynaptic AVA neurons to direct SPR, and is enriched in AVA neurites in the region of synapse formation with PHB neurons. Finally, we find that *clr-1/RPTP* acts in the same pathway as *unc-6/Netrin* and *unc-40/DCC* to mediate SPR. Our findings demonstrate a new role for *clr-1/RPTP* in SPR, and indicate that these three conserved families of proteins can act together to mediate communication between cells, which may provide insight into their roles in neurological disorders [13, 14] and cancer biology [15–17].

## Results

### *clr-1/RPTP* is required for formation of synapses between PHB and AVA neurons

To discover molecules that might act with UNC-6/Netrin and UNC-40/DCC in SPR, we introduced the NLG-1 GRASP marker into a series of strains with mutations in molecules that have neuronal function and/or expression. We found that in *clr-1* mutants, cell migration, axon guidance, and contact between PHB and AVA neurites appear normal (Fig 1E and 1F), but NLG-1 GRASP intensity is severely reduced, indicating a reduction in synapse formation (Fig 1G and 1H). We tested three loss-of-function alleles of *clr-1* and all showed significantly reduced NLG-1 GRASP intensity (Fig 1H). *clr-1* encodes a receptor protein tyrosine phosphatase (RPTP) with immunoglobulin-like and fibronectin III domains, similar to the Leukocyte common Antigen-Related protein (LAR) family (type IIa) RPTPs [12, 18]. In *clr-1/RPTP(e1745)* mutants, PHB axon length is slightly reduced, but other mutants with similarly reduced axon length, such as *ced-10/Rac1(n1993)*, have normal NLG-1 GRASP intensity, indicating that a slight reduction in PHB-AVA contact is unlikely to result in decreased synapse formation (S1A and S1B Fig). *clr-1/RPTP* promotes proper extension of highly branched PVD dendrites [19]. However, the extension of the AVA neurites was not decreased in *clr-1/RPTP* mutants (S1C and S1D Fig).

When PHB-AVA SPR is disrupted, PHB circuit function should be compromised. We tested this using a PHB circuit-specific behavioral assay. PHB sensory neurons mediate the nematode’s avoidance of sodium dodecyl sulfate (SDS) [4]. Wild-type animals will move backward into a control buffer for over one second, but stop backward movement into diluted SDS in approximately one third that time [10]. We compare these times using a response index (RI), dividing the average time backing into SDS by the average time backing into control buffer, and normalizing wild-type to 100%. A larger RI indicates impaired PHB circuit function [10]. *clr-1/RPTP* mutants have a severe defect in SDS avoidance, indicating that PHB circuit function is disrupted (Fig 1I). This defect in neural circuit function also indicates that reduction of NLG-1 GRASP fluorescence cannot be explained simply by a reduction in marker expression. However, to control for this possibility in another way, we also drove expression of GFP in PHB neurons and mCherry in AVA neurons in wild-type and *clr-1/RPTP* mutant animals with the same promoters used in the PHB-to-AVA NLG-1 GRASP marker, and measured average fluorescence intensity in the posterior region of AVA and the anterior region of PHB neurites. Expression levels were comparable between wild-type and *clr-1* in both pre- and postsynaptic neurons, indicating that the reduction in NLG-1 GRASP fluorescence is not the result of a reduction in marker expression in either cell (S2A Fig). We previously found that *nlg-1* mutants respond to SDS normally [10], thus impaired *nlg-1/Neuroligin 1* expression or localization cannot explain the defect in PHB circuit function observed in *clr-1/RPTP* mutant animals.

To determine if synaptic defects in *clr-1/RPTP* mutants are due to a defect in trafficking synaptic components to the distal synaptic region of PHB and AVA neurites, or if these defects might be due to a later recognition defect in SPR, we examined levels of synaptic components in the distal region of the neurites. Levels of the presynaptic vesicle marker RAB-3::mCherry and the presynaptic active zone marker GFP::ELKS-1 in PHB, and the postsynaptic marker NLG-1::YFP in AVA, are not reduced in *clr-1/RPTP* mutants (S2B–S2N Fig). This is similar to observations in *unc-6/Netrin* and *unc-40/DCC* mutants, and consistent with a role for *clr-1/RPTP* in SPR [10].

### *clr-1/RPTP* functions in AVA neurons to mediate SPR

**Fig 2:**
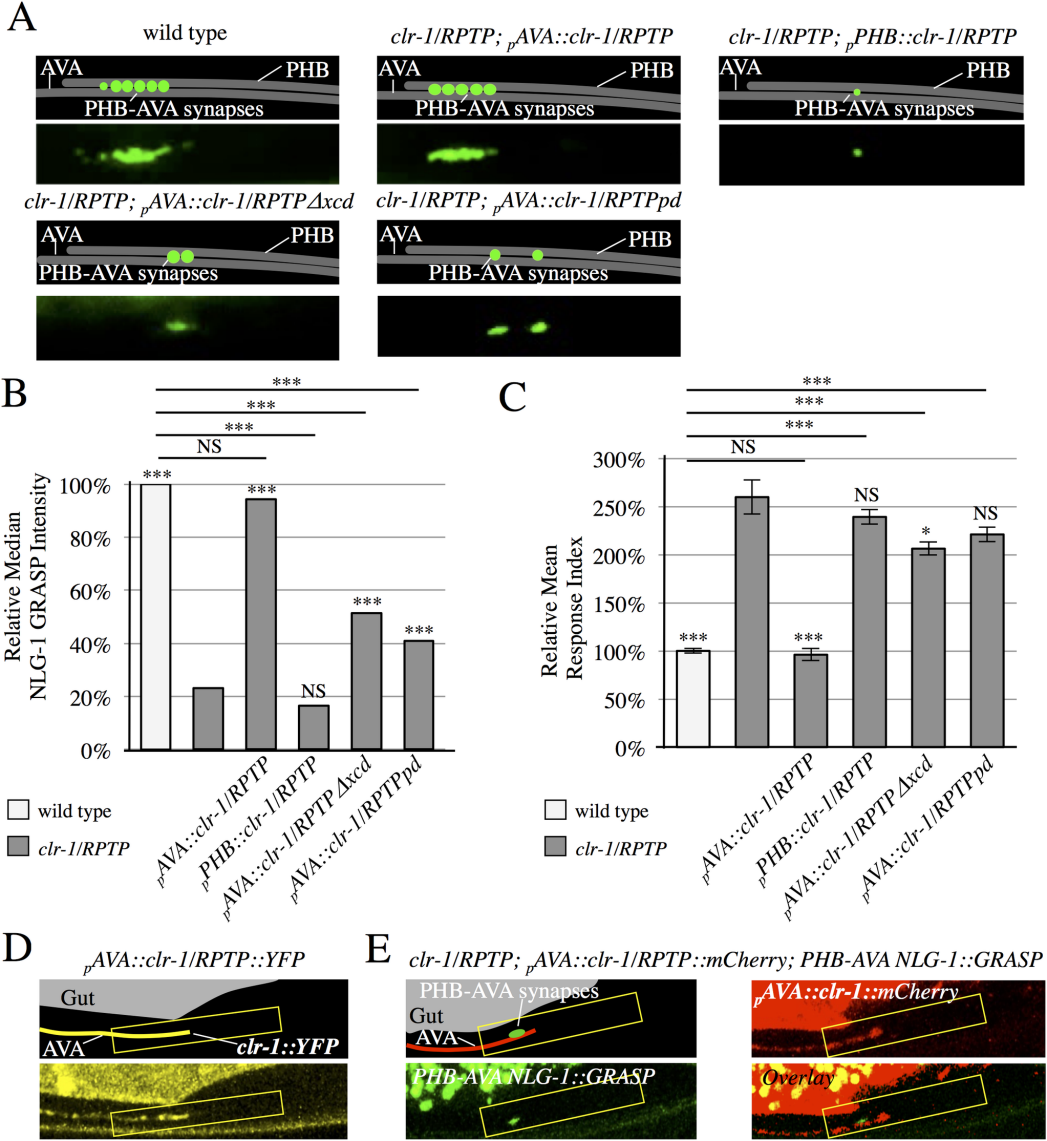
CLR-1/RPTP acts in postsynaptic neurons, and is localized to the synaptic region. (A) Schematics and micrographs of normal PHB-AVA NLG-1 GRASP fluorescence in wild-type and *clr-1/RPTP(e1745)* mutant animals expressing a transgene that drives expression of the *clr-1* cDNA in AVA neurons (_*p*_*AVA*::*clr-1/RPTP*), and reduced PHB-AVA NLG-1 GRASP fluorescence in *clr-1/RPTP(e1745)* mutant animals expressing either a construct that drives expression of the *clr-1/RPTP* cDNA in PHB neurons (_*p*_*PHB*::*clr-1/RPTP*), a transgene that drives expression of the *clr-1/RPTP* cDNA with the extracellular domain deleted in AVA neurons (_*p*_*AVA*::*clr-1/RPTPΔxcd)*, or a transgene that drives expression of the *clr-1/RPTP* cDNA with a mutation that inactivates the phosphatase domain (_*p*_*AVA*::*clr-1/RPTPpd*). (B) Quantification of NLG-1 GRASP fluorescence. Expression of *clr-1/RPTP* in AVAs, but not PHBs restores NLG-1 GRASP fluorescence in *clr-1/RPTP(e1745)* mutants (n>75). Expression of the *clr-1/RPTP* cDNA with the extracellular domain deleted or with a mutation in the active site of the phosphatase domain does not fully restore NLG-1 GRASP fluorescence in *clr-1/RPTP(e1745)* mutants (n>100). Two or more lines were examined with each transgene, and combined in the graph above. Values for each individual transgenic line are included in S2 Table. NS, not significant, ***P<0.001, *P<0.05, U-test. Comparison to *clr-1/RPTP* indicated over individual bars. *P*-values were adjusted for multiple comparisons using the Hochberg method. 95% confidence intervals for the medians are included in S1 Table. (C) Expression of *clr-1/RPTP* in AVAs, but not PHBs, rescues the behavioral defect in *clr-1/RPTP(e1745)* mutants (n>75). Expression of *clr-1/RPTP* cDNA with the extracellular domain deleted or with a mutation in the active site of the phosphatase domain does not fully rescue the behavioral defect in *clr-1/RPTP(e1745)* mutants (n≥60). NS, not significant, ***P<0.001, t-test. Comparison to *clr-1/RPTP* indicated over individual bars. *P*-values were adjusted for multiple comparisons using the Hochberg method. Error bars are SEM. (D) Schematic and micrograph of an animal expressing the *clr-1/RPTP* cDNA linked to *YFP* in AVA (_*p*_*AVA*::*clr-1/RPTP*::*YFP*). (E) Schematic and micrograph of an animal expressing the *clr-1/RPTP* cDNA linked to *mCherry* in AVA (_*p*_*AVA*::*clr-1/RPTP*::*mCherry*) and PHB-AVA NLG-1 GRASP, and overlay in a *clr-1/RPTP* mutant animal. (D-E), CLR-1 localization is brightest in the preanal ganglion (yellow box), and the majority of animals show localization in the anterior half of this region, where PHB-AVA synapses usually form (green fluorescence).

To determine if *clr-1/RPTP* was expressed in PHB or AVA neurons, we conducted expression studies utilizing a _*p*_*clr-1*::*GFP* transcriptional fusion transgene in wild-type animals. GFP expression was observed in AVA neurons, but not in PHB neurons (S3 Fig). To test if *clr-1/RPTP* can function in AVA neurons to promote PHB-AVA SPR, we generated a construct driving the expression of the *clr-1* cDNA under control of an AVA-selective promoter (_*p*_*AVA*::*clr-1*) and introduced it into *clr-1/RPTP* mutants. NLG-1 GRASP intensity and the behavioral output of the PHB circuit were rescued to wild-type levels in these transgenic animals, indicating that *clr-1/RPTP* can function in AVA neurons to direct SPR. Expression of the *clr-1* cDNA in PHB neurons did not rescue NLG-1 GRASP intensity or the behavioral output of the PHB circuit in *clr-1/RPTP* mutants, consistent with a role for *clr-1/RPTP* in AVA neurons (Fig 2A–2C).

To test if the extracellular domain of *clr-1/RPTP* is necessary for SPR, we generated a construct in which the extracellular domain of the *clr-1/RPTP* cDNA was deleted and expressed under the direction of an AVA-selective promoter (_*p*_*AVA*::*clr-1/RPTPΔxcd*), and introduced it into *clr-1/RPTP* mutants. Unlike the full-length *clr-1/RPTP* cDNA under the direction of the AVA promoter, _*p*_*AVA*::*clr-1/RPTPΔxcd* did not rescue either NLG-1 GRASP fluorescence or the behavioral output of the circuit to wild-type levels; NLG-1 GRASP fluorescence was 51% of wild-type levels, and the relative response index was 206% of wild-type levels (Fig 2A–2C). This indicates that the extracellular domain is required for full SPR function. To determine if the phosphatase activity of *clr-1/RPTP* is required for PHB-AVA SPR, we introduced a point mutation into the _*p*_*AVA*::*clr-1/RPTP* construct that inactivates the membrane-proximal phosphatase domain [18] (_*p*_*AVA*::*clr-1/RPTPpd*). Again, the NLG-1 GRASP intensity and behavioral output of the PHB circuit were not rescued to wild-type levels; NLG-1 GRASP fluorescence was 41% of wild-type levels, and the relative response index was 221% of wild-type levels (Fig 2A–2C). This indicates that *clr-1/RPTP* phosphatase activity is required for full SPR function as well.

To better understand the mechanism by which CLR-1/RPTP directs SPR, we visualized the subcellular localization of CLR-1/RPTP in AVA neurons. We generated a translational fusion of the *clr-1/RPTP* cDNA to *YFP* under the direction of an AVA-selective promoter *(*_*p*_*AVA*::*clr-1/RPTP*::*YFP)*. We found that CLR-1/RPTP was localized throughout the AVA axon, but was brightest in the preanal ganglion, where AVA neurons contact PHB neurons. Interestingly, within the region of the preanal ganglion, it was usually concentrated in the anterior half, where the majority of synapses between PHB and AVA normally form (Fig 2D). We generated a similar translational fusion of the *clr-1/RPTP* cDNA to *mCherry (*_*p*_*AVA*::*clr-1/RPTP*::*mCherry)* and introduced it into animals expressing PHB-AVA NLG-1 GRASP. We similarly found that the NLG-1 GRASP signal was usually confined to the anterior half of the preanal ganglion where CLR-1/RPTP localized (Fig 2E). This localization is consistent with a role for CLR-1/RPTP in SPR.

### *clr-1/RPTP* function in SPR begins in late embryogenesis

**Fig 3:**
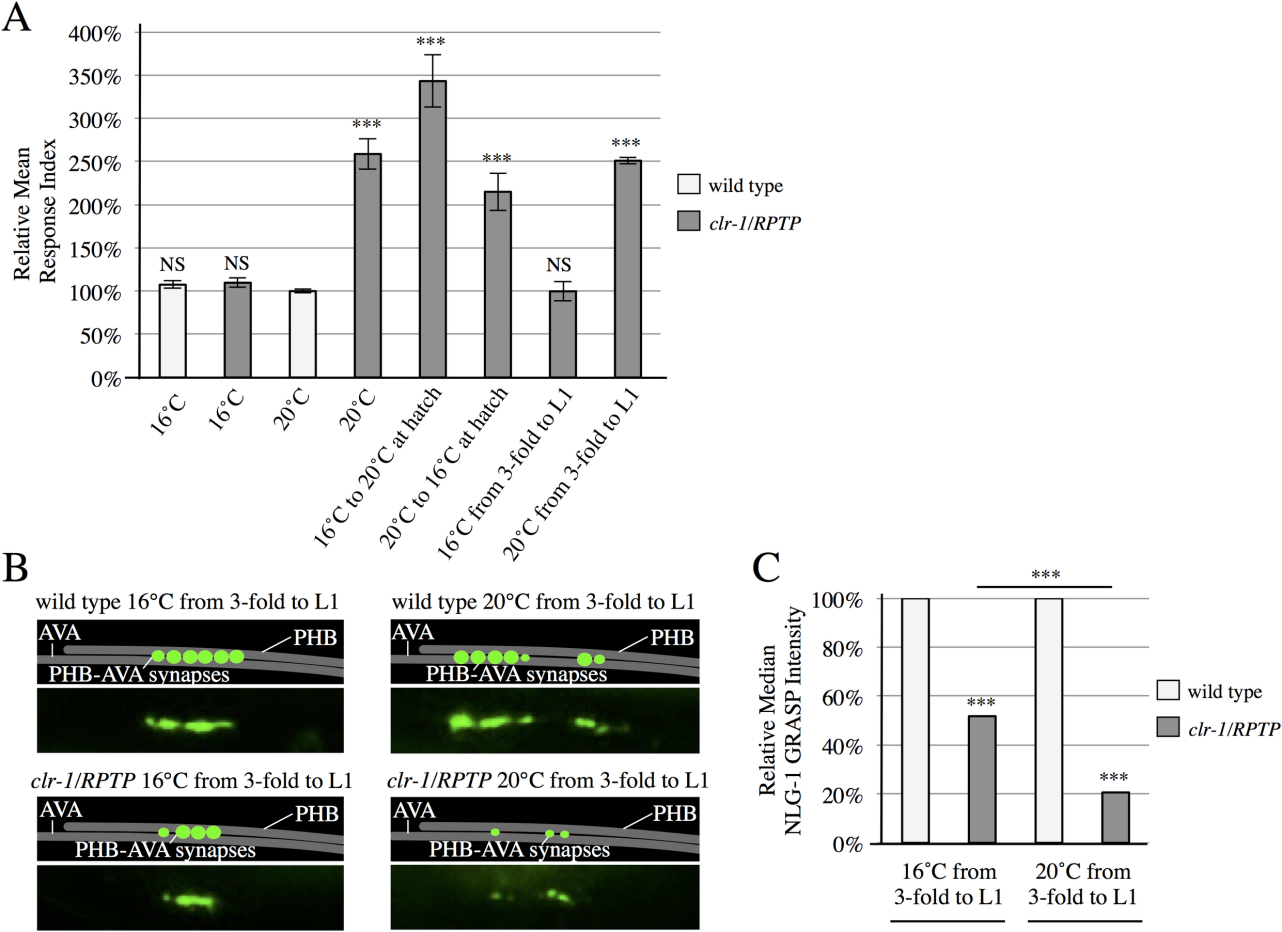
CLR-1/RPTP is required during late embryogenesis and the first larval stage. (A) *clr-1/RPTP(e1745)* animals shifted from the permissive temperature (16°C) to the restrictive temperature (20°C) or vice versa after hatching display defective behavior (n≥40). *clr-1/RPTP(e1745)* animals at 16°C during the 3-fold embryo and larval stage 1 (L1) respond normally to SDS. Animals at 20°C during these stages have defective responses (n>65). NS, not significant, ***P<0.001, t-test, comparison to wild-type. *P*-values were adjusted for multiple comparisons using the Hochberg method. Error bars are SEM. (B) Schematics and micrographs of wild-type and *clr-1/RPTP* animals kept at 20°C or 16°C during the 3-fold embryo and L1 stages. (C) Quantification of reduced NLG-1 GRASP fluorescence intensity in *clr-1/RPTP* animals kept at 20°C during these stages, in comparison with *clr-1/RPTP* animals kept at 16°C during these stages. ***P<0.001, U-test, comparison to wild-type if directly over bar, or as indicated. *P*-values were adjusted for multiple comparisons using the Hochberg method. 95% confidence intervals for the medians are included in S1 Table.

CLR-1/RPTP could function in embryogenesis during establishment of correct SPR, later to maintain proper synaptic connectivity, or at both times. To test when CLR-1/RPTP functions, we took advantage of the temperature-sensitive nature of the *clr-1/RPTP(e1745)* allele [18, 20]. Interestingly, behavioral function was impaired in *clr-1(e1745)* animals placed at the restrictive temperature only during embryogenesis, or placed at the restrictive temperature only after embryogenesis, indicating that *clr-1/RPTP* function is required both during and after embryogenesis (Fig 3A). To test when *clr-1/RPTP* function is necessary and sufficient, we shifted the animals immediately before the last stage of embryogenesis (the 3-fold embryo) and after the first larval stage (L1). Animals placed at the restrictive temperature only during the 3-fold embryo and L1 stages exhibited impaired behavior, while animals placed at the permissive temperature during the same stages exhibited normal behavior (Fig 3A). This indicates that *clr-1/RPTP* function is necessary during the 3-fold embryo and L1 stages, the period when synaptogenesis is likely initiated and the stage directly after, consistent with a role in SPR.

In addition, we observed NLG-1 GRASP fluorescence intensity in animals placed at the permissive or restrictive temperature during the 3-fold embryo and L1 stages. We observed a reduction in NLG-1 GRASP fluorescence intensity in *clr-1/RPTP* animals shifted from the restrictive to the permissive temperature at the 3-fold embryo stage, and shifted from the permissive to the restrictive temperature at the end of the L1 stage, possibly due to stress on the mutant animals from multiple temperature shifts. Even so, NLG-1 GRASP intensity was severely and significantly reduced from this level in animals moved to the restrictive temperature for the 3-fold embryo and L1 stages, compared with animals kept at the permissive temperature during the same periods (Fig 3B and 3C). This is also consistent with a function during the 3-fold and L1 stages.

### Overexpression of *clr-1/RPTP* in AVA neurons is sufficient to trigger additional synaptogenesis

**Fig 4:**
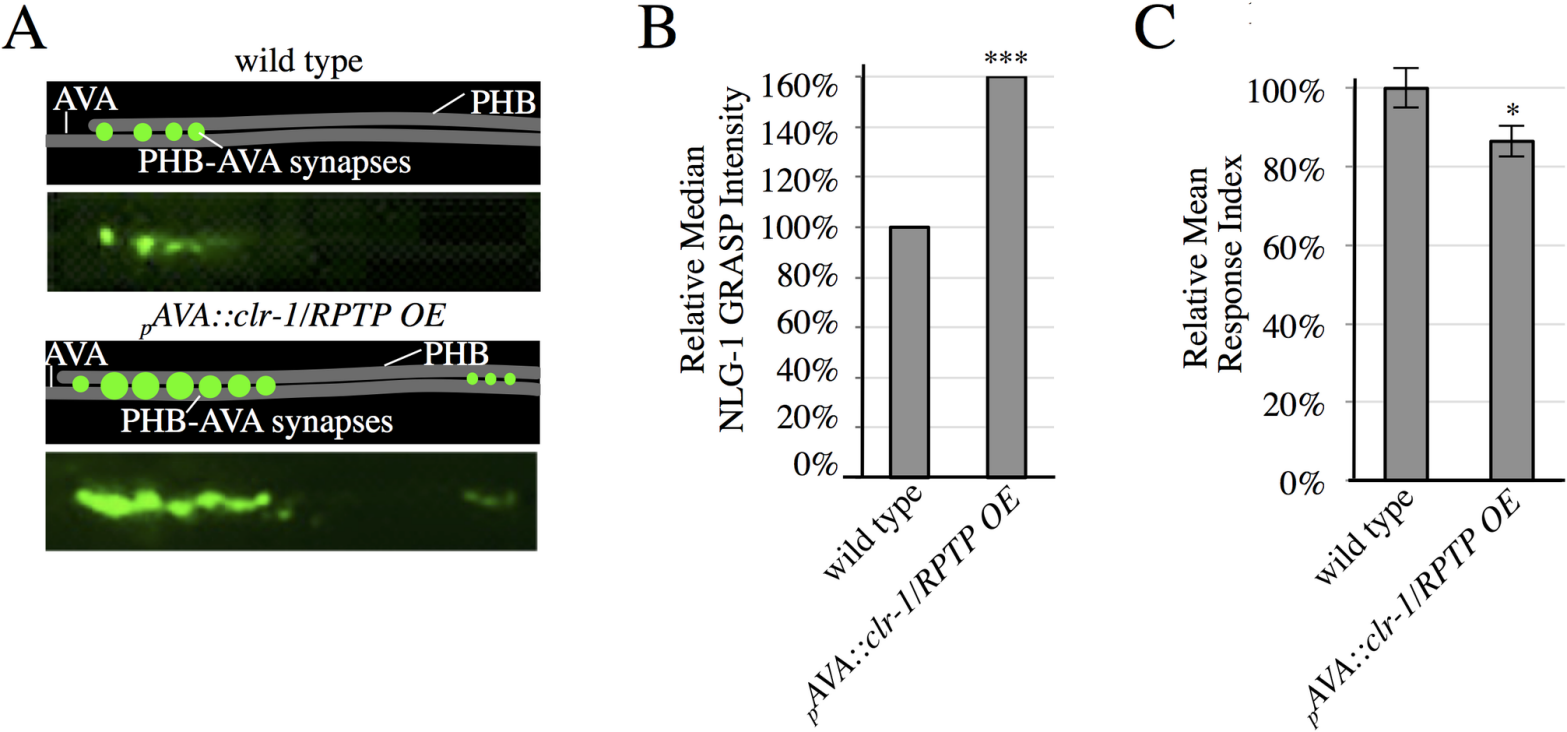
CLR-1/RPTP is sufficient to drive increased synaptogenesis. (A) Representative schematics and micrographs of NLG-1 GRASP fluorescence labeling PHB-AVA synapses in wild-type animals and animals overexpressing *clr-1/RPTP* in AVA neurons (_*p*_*AVA*::*clr-1/RPTP OE)*. (B) Quantification of NLG-1 GRASP fluorescence. Overexpression of *clr-1/RPTP* in AVA neurons results in increased NLG-1 GRASP fluorescence (n>100). Two lines were examined with this transgene, and combined in the graph above. Values for each individual transgenic line are included in S2 Table. ***P<0.001, U-test, comparison to wild-type. 95% confidence intervals for the medians are included in S1 Table. (C) Overexpression of *clr-1/RPTP* in AVAs results in a faster behavioral response (n = 80). *P<0.05, t-test, comparison to wild-type. Error bars are SEM.

If CLR-1/RPTP functions in the SPR signaling event between PHB and AVA neurons, increasing the expression of *clr-1/RPTP* in AVA neurons should direct additional synapse formation between these neurons. In fact, overexpression (OE) of _*p*_*AVA*::*clr-1/RPTP* was sufficient to drive a significant increase in PHB-AVA synaptogenesis (Fig 4A and 4B). This is similar to the increase in PHB-AVA synaptogenesis observed in animals overexpressing *unc-6/Netrin* in AVA neurons [10], and demonstrates an ability to drive synaptogenesis. To test if these synapses were functional, we also conducted behavioral analysis. We found that the response index was significantly smaller, consistent with potentiation of the circuit (Fig 4C).

### *clr-1/RPTP* acts with *unc-6/Netrin* and *unc-40/DCC* to mediate SPR

**Fig 5:**
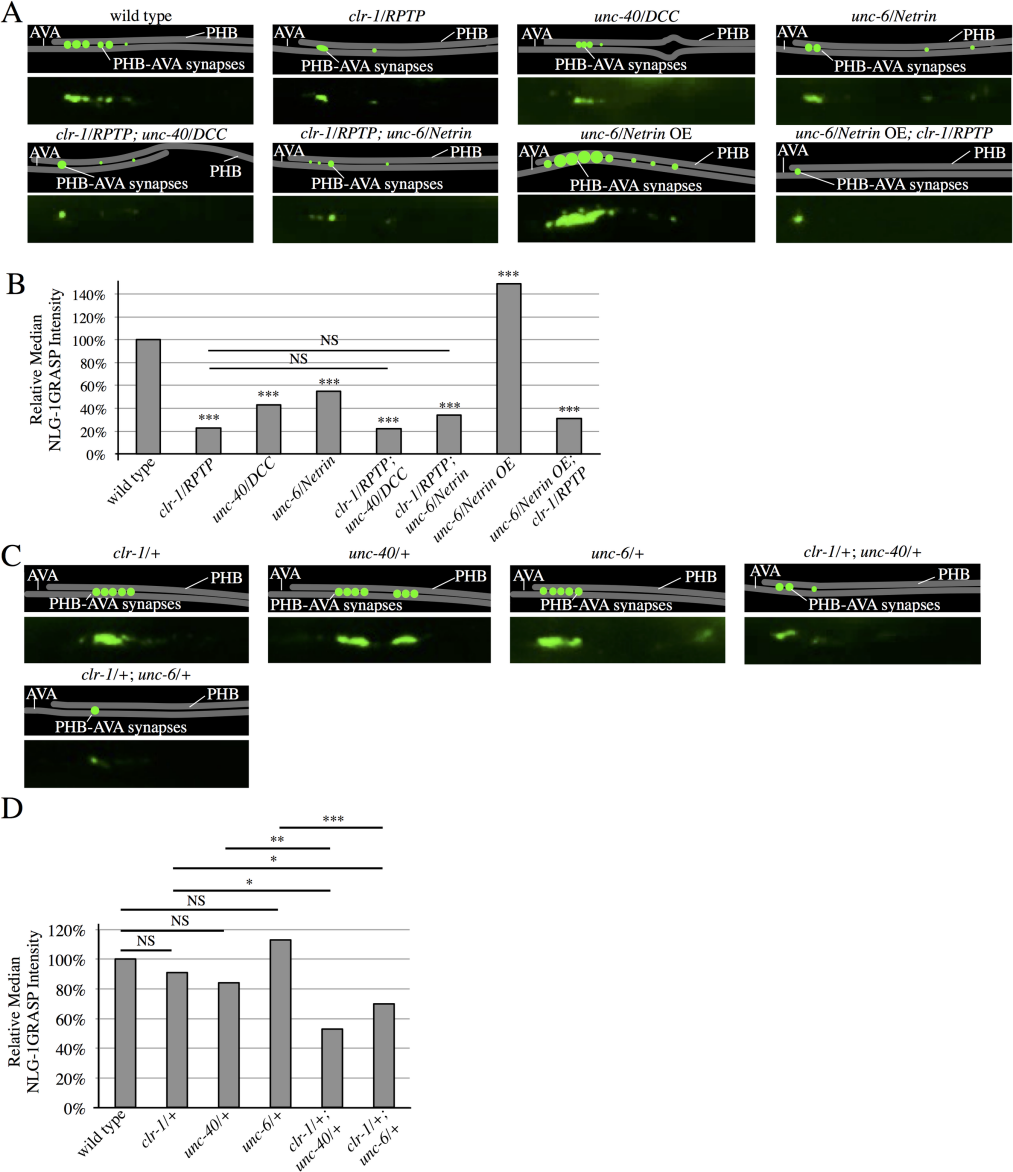
CLR-1/RPTP acts in the UNC-6/Netrin and UNC-40/DCC SPR pathway. (A) Representative diagrams and micrographs of NLG-1 GRASP fluorescence labeling PHB-AVA synapses in wild-type, *clr-1/RPTP(e1745)*, *unc-40/DCC(e271)*, *unc-6/Netrin(ev400)*, *clr-1/RPTP; unc-40/DCC*, *clr-1/RPTP; unc-6/Netrin*, *unc-6/NetrinOE*, and *unc-6/NetrinOE; clr-1/RPTP* mutants. (B) Quantification of a reduction in NLG-1 GRASP fluorescence in *clr-1/RPTP(e1745)*, *unc-40/DCC(e271)*, *unc-6/Netrin(ev400)*, *clr-1/RPTP; unc-40/DCC*, *clr-1/RPTP; unc-6/Netrin*, and *unc-6/Netrin OE; clr-1/RPTP* animals, and an increase in NLG-1 GRASP fluorescence in *unc-6/Netrin OE* animals (n>30). ***P<0.001, U-test, comparison to wild-type. *P*-values were adjusted for multiple comparisons using the Hochberg method. 95% confidence intervals for the medians are included in S1 Table. (C) Representative diagrams and micrographs of normal levels of NLG-1 GRASP fluorescence labeling PHB-AVA synapses in *clr-1* heterozygotes (*clr-1/+)*, *unc-40/DCC* heterozygotes (*unc-40/+)*, and *unc-6/Netrin* heterozygotes (*unc-6/+)*, and reduced NLG-1 GRASP fluorescence in trans-heterozygotes with *clr-1/RPTP* and *unc-40/DCC* (*clr-1/+; unc-40/+*), and trans-heterozygotes with *clr-1/RPTP* and *unc-6/Netrin* (*clr-1/+; unc-6/+)*. (D) Quantification of NLG-1 GRASP intensity that is not statistically significantly different from wild-type in *clr-1/+*, *unc-40/+*, and *unc-6/+*, and reduced NLG-1 GRASP fluorescence intensity in *clr-1/+; unc-40/+* and *clr-1/+; unc-6/+* animals. NS, not significant, ***P<0.001, **P<0.01, *P<0.05, U-test. 95% confidence intervals for the medians are included in S1 Table.

To test if UNC-6/Netrin and UNC-40/DCC act with CLR-1/RPTP in promoting SPR, we generated double-mutants between *clr-1/RPTP* and *unc-40/DCC*, and *clr-1/RPTP* and *unc-6/Netrin*. These double-mutants did not have more severe SPR defects when compared with *clr-1* single mutants, indicating that *unc-40/DCC*, *unc-6/Netrin*, and *clr-1/RPTP* function in the same SPR pathway (Fig 5A and 5B). If *clr-1/RPTP* receives the *unc-6/Netrin* signal, *clr-1/RPTP* should act downstream of *unc-6/Netrin*. To test this, we introduced the *clr-1/RPTP(e1745)* mutation into animals overexpressing *unc-6/Netrin* to determine if the high levels of synaptogenesis were suppressed. Indeed, PHB-AVA synapses were dramatically reduced (Fig 5A and 5B), consistent with a role for *clr-1/RPTP* downstream of *unc-6/Netrin* in SPR. In addition, we generated trans-heterozygotes between the recessive *clr-1/RPTP* and *unc-40/DCC* mutants, and *clr-1/RPTP* and *unc-6/Netrin* mutants. NLG-1 GRASP fluorescence was significantly reduced in both trans-heterozygous strains, indicating that these genes likely function together in SPR (Fig 5C and 5D).

### *clr-1/RPTP* is required for formation of synapses between AVA and postsynaptic neurons

To determine if *clr-1/RPTP* affects other synaptic connections, we introduced the *clr-1/RPTP* mutation into a NLG-1 GRASP marker labeling synapses between AVA neurons and their postsynaptic partners, the VA and DA motorneurons [11]. Specifically, we assayed synapses between AVA and VA10 motorneurons. A cluster of AVA-VA10 synapses is localized between the VA10 and DA7 neurons in wild-type animals [11, 21], but was lost or reduced in most *clr-1/RPTP* mutants (S4 Fig). This indicates that SPR defects in *clr-1/RPTP* mutants are not specific to PHB-AVA synapses, and that *clr-1/RPTP* has broader functions in synaptic partner recognition.

## Discussion

Here, we demonstrate a novel role for *clr-1/RPTP* in promoting SPR between neurons in complex *in vivo* environments. In *clr-1/RPTP* mutants, fluorescence of the NLG-1 GRASP marker labeling synapses between PHB sensory neurons and AVA interneurons is severely reduced, indicating a reduction of synapses between these neurons. In addition, a PHB circuit-specific behavioral response is compromised, consistent with a loss of synaptic function. A transcriptional fusion of *clr-1/RPTP* showed expression in AVA, but not PHB neurons, and expression of *clr-1/RPTP* in AVA neurons was sufficient to rescue the *clr-1/RPTP* mutant defects, indicating a postsynaptic role. Deletion of the *clr-1/RPTP* extracellular domain or a mutation in the catalytic site of the phosphatase domain compromises this rescue, indicating that both domains are required for full SPR activity. Overexpression of *clr-1/RPTP* in AVA neurons results in increased NLG-1 GRASP fluorescence and potentiates circuit function, suggesting that postsynaptic *clr-1/RPTP* is sufficient to promote synaptogenesis. Our genetic analysis indicates that *clr-1/RPTP* acts in the same SPR pathway as *unc-6/Netrin* and *unc-40/DCC*. *clr-1/RPTP* is also required for synaptogenesis between AVA and its postsynaptic partner, the VA10 motorneuron, indicating that *clr-1/RPTP* may have a broader role in SPR between other neurons.

Our work describes the first role in synaptogenesis for *clr-1*, and the first postsynaptic role in synaptogenesis for a LAR family member in *C*. *elegans*. The LAR family member *ptp-3* has also been studied in *C*. *elegans*. *ptp-3* isoform A is presynaptic and required for proper presynaptic morphology at neuromuscular junctions, indicating a role in presynaptic assembly [22, 23]. In *Drosophila* and vertebrate systems, LAR family members have also been found to regulate synaptogenesis, although the focus of most work has been on presynaptic roles for these proteins [24–26]. Yet, LAR has also been found at postsynaptic sites in vertebrate systems [27–29]. PHB-AVA synapses can be visualized and their circuit function tested in intact, live animals, in a genetically tractable system, making them a powerful system in which to understand the roles of LAR family members in postsynaptic cells, and identify their interactors.

LAR family members can act with molecules in the same cell and in their presumptive synaptic partners to mediate synapse formation. Studies in invertebrate and vertebrate systems have demonstrated that one mechanism by which LARs can promote presynaptic assembly is via their interaction with liprins [30–32]. Liprin-α interacts with several other active zone proteins, providing a mechanism by which presynaptic components may be recruited to sites of LAR binding [26, 32]. LAR family RPTPs can act in *trans* with several proteins including Netrin-G ligand, IL1RAPL, neurotrophin receptor tyrosine kinase C, Slit- and Trk-like proteins, and synaptic adhesion-like molecule 3, and in *cis* with the heparin sulfate proteoglycans glypican and syndecan [33–40]. Work in cultured cells demonstrates that the Netrin family member Netrin-G1 can interact *in cis* with LAR to mediate synaptogenesis [41]. This is similar to what we observe at PHB-AVA synapses, where both UNC-6/Netrin and CLR-1/RPTP function in the same cells, and genetic evidence shows that they act in the same pathway to promote SPR. However, in cultured cells, the receptor on the opposing cell is the Netrin G Ligand NGL-1 [41]. In PHB-AVA SPR, we find that the receptor functioning in the opposing partner is Netrin’s canonical receptor DCC. CLR-1/RPTP, UNC-6/Netrin and UNC-40/DCC have previously been shown to act together in mechanosensory neuron axon guidance. However, rather than promoting UNC-40/DCC function in *trans* as in SPR, CLR-1/RPTP acts in *cis* to negatively regulate UNC-40/DCC function or the function of downstream signaling molecules [12]. Thus, this study defines a new mechanism by which synaptic partner recognition is mediated.

**Fig 6:**
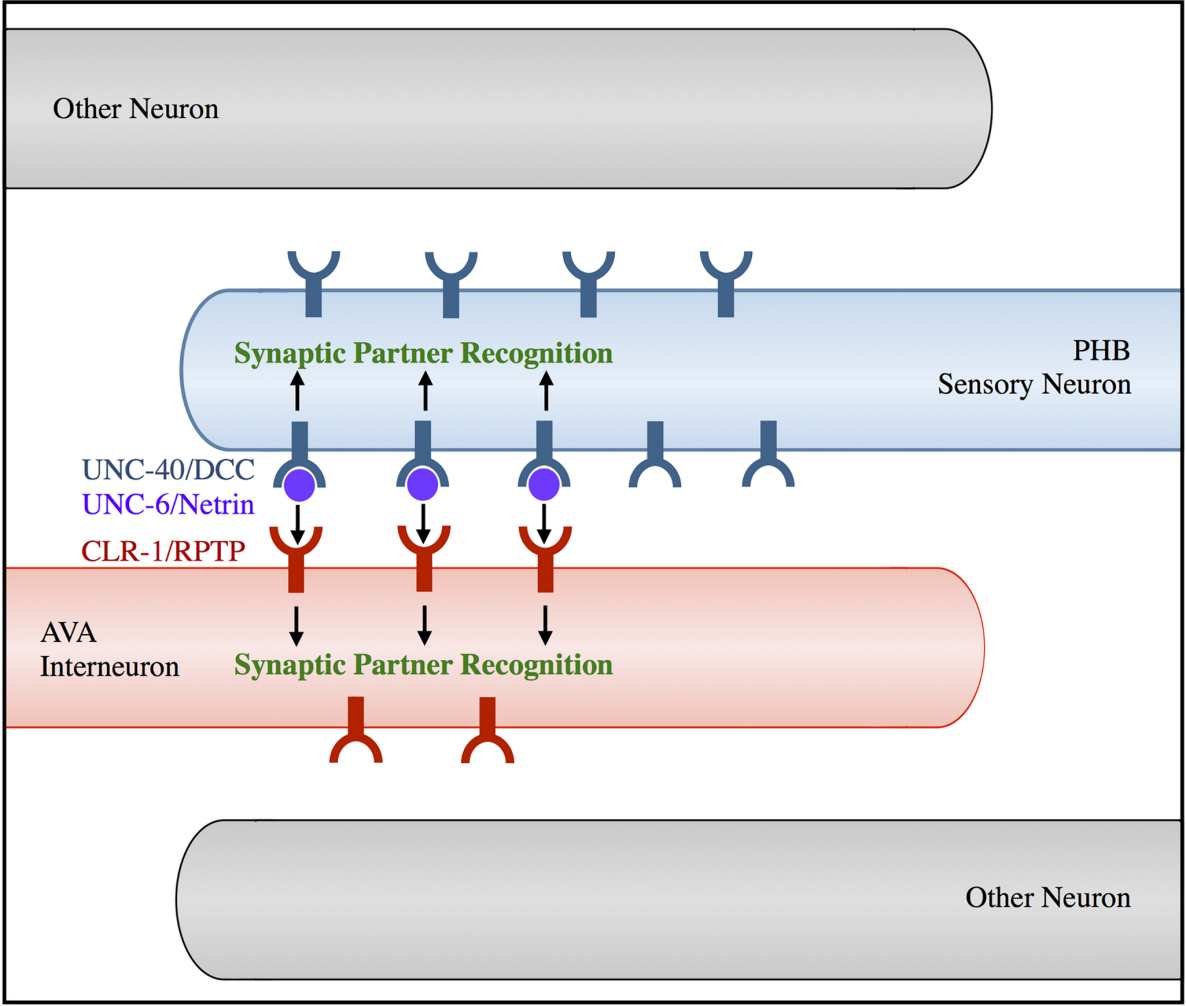
CLR-1/RPTP acts in the UNC-6/Netrin and UNC-40/DCC pathway to direct SPR. In this model, limiting amounts of UNC-6/Netrin secreted from AVA interneurons bind UNC-40/DCC expressed in PHB neurons. CLR-1/RPTP expressed in AVA neurons acts genetically downstream of UNC-6/Netrin in the SPR pathway, and its extracellular domain is required for full SPR function, suggesting that it may interact with UNC-6/Netrin, UNC-40/DCC, or another unidentified ligand or receptor. The requirement of CLR-1/RPTP’s phosphatase domain for rescue indicates that phosphatase activity is also required for its function in SPR.

Our previous work suggests a model in which limiting amounts of UNC-6/Netrin are secreted from postsynaptic AVA neurons, binding UNC-40/DCC in presynaptic neurons to specify them as the correct presynaptic partners [10]. Our current work indicates CLR-1/RPTP likely transduces the SPR signal into presumptive postsynaptic AVA neurons, promoting synaptogenesis. We propose that CLR-1/RPTP may interact in *trans* with UNC-6/Netrin, UNC-40/DCC, or a ligand or receptor that is yet to be identified, to generate PHB-AVA SPR (Fig 6). The requirement of the *clr-1/RPTP* extracellular domain for full rescue of the *clr-1/RPTP* defect is consistent with this model. The small degree of SPR rescue is consistent either with a low level of CLR-1/RPTP activation in the absence of its extracellular domain, or by a minority of CLR-1/RPTP synaptogenic activity not requiring a *trans*-interaction. CLR-1/RPTP requires phosphatase activity for the majority of its function in SPR. Although phosphatase activity of *Drosophila* LAR is not required for photoreceptor axon targeting [42] or viability [43], LAR phosphatase activity is required for many processes, including growth of neuromuscular junctions [40]. Several LAR family substrates have been identified, including N-cadherin, β-catenin, Abelson kinase, Enabled, Trio, p250RhoGAP, and multiple tyrosine kinases, and their regulation by LAR may modulate synaptic adhesion and actin dynamics [44].

This study defines a new pathway by which SPR is governed. The ability of CLR-1/RPTP to function in postsynaptic cells with secreted UNC-6/Netrin and presynaptic UNC-40/DCC also demonstrates a new mode of action for these conserved molecules. The distinct molecular pathways and sites of action discovered for LAR family members may allow these same molecules to govern different processes in neural circuit formation and other developmental processes. Understanding how Netrin, DCC, and LAR-RPTP family proteins act together to regulate cell-cell signaling may be important for human health, as these genes are associated with neurological disorders, such as schizophrenia [13, 14], as well as cancer [16, 17, 45].

## Materials and methods

### Strains and genetics

All worms were maintained according to standard protocols [46] and were raised on 6 cm NGM plates seeded with OP50 *Escherichia coli* at 20°C, except for the worms used for the temperature shift experiment, which were raised at 16°C when noted. Wild-type strains were *C*. *elegans* variety Bristol, strain N2. Mutants used for this study include *clr-1(e1745) II*, *clr-1(e2530) II*, *clr-1(n1992) II*, [18, 20], *unc-6(ev400) X* [47], *unc-40(e271) I* [20] and *ced-10(n1993) IV* [48]. Except for strains containing *wyEx1364*, *wyEx1402*, *iyEx323*, and *iyIs8* (see below), all strains contain the integrated PHB-AVA NLG-1 GRASP marker *wyIs157 IV* (*pSM*::_*p*_*gpa-6*::*nlg-1*::*spGFP1-10* (60 ng/μl), *pSM*::_*p*_*flp-18*::*nlg-1*::*spGFP11* (30 ng/μl), *pSM*::_*p*_*nlp-1*::*mCherry* (10 ng/μl), *pSM*::_*p*_*flp-18*::*mCherry* (5 ng/μl) and _*p*_*odr-1*::*DsRed2* (20 ng/μl)) [10].

Transgenic lines used in this study include lines carrying _*p*_*AVA*::*clr-1/RPTP* for both cell-specific rescue of *clr-1(e1745) II* (Fig 2A–2C), and overexpression in AVA neurons (Fig 4A–4C): *iyEx97* (*pSM*::_*p*_*rig-3*::*clr-1* (40 ng/μl), _*p*_*unc-122*::*RFP* (20 ng/μl)) and *iyEx101* (*pSM*::_*p*_*rig-3*::*clr-1* (60 ng/μl), _*p*_*unc-122*::*RFP* (20 ng/μl)). Transgenic lines carrying _*p*_*PHB*::*clr-1/RPTP* include *iyEx364*, *iyEx365*, and *iyEx366* (*pSM*::_*p*_*nlp-1*::*clr-1* (1–5 ng/μl), _*p*_*unc-122*::*RFP* (22 ng/μl)) (Fig 2A–2C). Transgenic lines carrying _*p*_*AVA*::*clr-1/RPTPΔxcd* include *iyEx133*, *iyEx134*, *iyEx135*, and *iyEx362* (*pSM*::_*p*_*rig-3*::*clr-1Δxcd*::*mCherry* (60 ng/μl), _*p*_*unc-122*::*RFP* (20 ng/μl)) (Fig 2A–2C). Transgenic lines carrying _*p*_*AVA*::*clr-1/RPTPpd* include *iyEx169*, *iyEx170* and *iyEx174* (*pSM*::*rig-3*::*clr-1pd*::*mCherry* (60 ng/μl), _*p*_*unc-122*::*RFP* (21 ng/μl)) (Fig 2A–2C). The transgenic line generated to determine the subcellular localization of CLR-1/RPTP in AVA neurons was *iyEx121* (*pSM*::_*p*_*rig-3*::*clr-1*::*YFP* (85 ng/μl), _*p*_*unc-122*::*RFP* (20 ng/μl)). The transgenic line generated to visualize CLR-1/RPTP co-localization with NLG-1 GRASP contained *iyEx175* (*pSM*::_*p*_*rig-3*::*clr-1*::*mCherry* (60 ng/μl), _*p*_*unc-122*::*RFP* (20 ng/μl)) and *iyIs2* (*pSM*::_*p*_*gpa-6*::*nlg-1*::*GFP1-10* (30 ng/μl), *pSM*::_*p*_*flp-18*::*nlg-1*::*GFP11* (15 ng/μl), _*p*_*odr-1*::*DsRed2* (50 ng/μl)), in a *clr-1(e1745)* background (Fig 2D and 2E). For overexpression of *unc-6/Netrin* in AVAs in wild-type and *clr-1/RPTP* mutant backgrounds, the transgenic line used was *iyEx47* [10] (Fig 5A and 5B).

For measurement of AVA neurites length, we generated the transgenic line *wyEx1364* (*pSM*::_*p*_*gpa-6*::*GFP* (50ng/μl), *pSM*::_*p*_*flp-18*::*mCherry* (10 ng/μl) and _*p*_*unc-122*::*RFP* (20 ng/μl)) (S1C and S1D Fig).

Transgenic lines used to assay expression levels from the promoters that drive PHB-AVA NLG-1 GRASP were *wyEx1402* (*pSM*::_*p*_*gpa-6*::*GFP* (50 ng/μl) and _*p*_*odr-1*::*DsRed2* (20 ng/ μl)) and *wyIs157* (S2A Fig). The transgenic lines used to visualize localization of presynaptic and postsynaptic markers in wild-type and *clr-1* mutant animals were *wyEx2309* for RAB-3, *iyEx82* for NLG-1, and *iyEx83* for ELKS-1 [10] (S2B–S2N Fig).

The transgenic lines generated to determine the expression pattern of *clr-1* in the posterior region was *iyEx61* (*pSM*::_*p*_*clr-1*::*GFP* (50ng/μl), _*p*_*unc-122*::*RFP* (20 ng/μl)) with *wyIs157*, and in the head was *iyEx323* (*pSM*::_*p*_*clr-1*::*GFP* (20 ng/μl), *pSM*::_*p*_*rig-3*::*mCherry* (17 ng/μl), _*p*_*unc-122*::*RFP* (22.5 ng/μl)) (S3 Fig).

To test if *clr-1/RPTP* is required for AVA-VA synapses, we stably integrated the *wyEx1845* AVA-VA/DA NLG-1 GRASP marker (*pSM*::_*p*_*unc-4*::*nlg-1*::*spGFP1-10* (20 ng/μl), *pSM*::_*p*_*flp-18*::*nlg-1*::*spGFP11* (30 ng/μl), *pSM*::_*p*_*unc-4*::*mCherry* (5 ng/μl), _*p*_*odr-1*::*DsRed2* (50 ng/μl)) [10] into the genome to generate *iyIs8* (S4 Fig).

### Cloning and constructs

Constructs were generated using standard molecular techniques. To generate *pSM*::_*p*_*rig-3*::*clr-1* (referred to as _*p*_*AVA*::*clr-1/RPTP* in the text), an N2 *C*. *elegans* cDNA library was generated by isolating *C*. *elegans* mRNA using Sigma Tri Reagent (TRIzol) to break the worm cuticle, chloroform to isolate RNA, and the Qiagen RNeasy Mini kit to purify the RNA. The Invitrogen SuperScript II Reverse Transcriptase kit was used to generate cDNA. *clr-1* cDNA was amplified using *clr-1*-specific primers (MVP281: AGACGTCGACATGCGAATAAATCGATGGATC and MVP282: TATTTGGTACCCTACCTATATGTCTTAGAGATA) that introduced the SalI and Acc65I restriction sites. The *clr-1* cDNA was subcloned into the SalI-Acc65I fragment from *pSM*::_*p*_*rig-3*, which was made by subcloning the *rig-3* promoter from *pSM*::_*p*_*rig-3*::*MT*::*unc-6* [10] using SphI and AscI restriction sites into the SphI-AscI fragment from *pSMΔ* (a gift from S. McCarroll).

To generate the *pSM*::_*p*_*clr-1*::*GFP* construct, (referred to as _*p*_*clr-1*::*GFP* in the text) the *clr-1* promoter (4480 bp upstream of the *clr-1* start site) was amplified from genomic DNA using _*p*_*clr-1* specific primers (MVP227: TAACGGCGCGCCGAGAATGAGGTTACGATCTAC and MVP228: ACATACCCGGGGTTTCCGCGTTAATTTAAAAGCC) that introduced AscI and SmaI restriction sites. Then, the _*p*_*clr-1* fragment was subcloned into the AscI-SmaI fragment from *pSM*::*GFP* (a gift from S. McCarroll).

To generate *pSM*::_*p*_*rig-3*::*clr-1*::*YFP* (referred to as _*p*_*AVA*::*clr-1/RPTP*::*YFP* in the text), the *clr-1* cDNA (without its stop codon) was amplified from *pSM*::_*p*_*rig-3*::*clr-1* using *clr-1* specific primers that introduced the SalI and Acc65I restriction sites (MVP344: TATTGGTACCCCTATATGTCTTAGAGATATAG and MVP281: AGACGTCGACATGCGAATAAATCGATGGATC). The *clr-1* cDNA was subcloned into the SalI-Acc65I fragment from *pSM*::_*p*_*rig-3*::*YFP*. *pSM*::_*p*_*rig-3*::*YFP* was made by amplifying the YFP fragment from *pSM*::_*p*_*rig-3*::*unc-6*::*YFP* [10] using primers that introduced a 10GS linker as well as the Acc65I and SacI restriction sites (MVP338: AGACGGTACCGGATCTGGATCTGGATCTGGATCTGGATCTATGAGTAAAGGAGAAGAACTT and MVP339: TATTGAGCTCCTATTTGTATAGTTCATCCATG). The 10GS linker-YFP fragments were then subcloned into the Acc65I-SacI fragment of *pSM*::_*p*_*rig-3*.

To generate *pSM*::_*p*_*rig-3*::*clr-1*::*mCherry* (referred to as _*p*_*AVA*::*clr-1/RPTP*::*mCherry* in the text), the *clr-1* cDNA (without its stop codon) was amplified from *pSM*::_*p*_*rig-3*::*clr-1* using *clr-1* specific primers that introduced the SalI and Acc65I restriction sites (MVP344: TATTGGTACCCCTATATGTCTTAGAGATATAG and MVP281: AGACGTCGACATGCGAATAAATCGATGGATC). The *clr-1* cDNA was subcloned into the SalI-Acc65I fragment from *pSM*::_*p*_*rig-3*::*mCherry*. *pSM*::_*p*_*rig-3*::*mCherry*, was made by amplifying mCherry from _*p*_*ttx-3*::*mCherry* [49] using primers that also introduced a 10GS linker and Acc65I and SacI restriction sites (MVP340: AGACGGTACCGGATCTGGATCTGGATCTGGATCTGGATCTATGGTCTCAAAGGGTGAAGA and MVP341: TATTGAGCTCCTTATACAATTCATCCATGCC). The *10GSlinker*::*mCherry* was then subcloned into the Acc65I-SacI fragment of *pSM*::_*p*_*rig-3 to generate pSM*::_*p*_*rig-3*::*mCherry*.

To generate *pSM*::_*p*_*rig-3*::*clr-1Δxcd*::*mCherry* (referred to as _*p*_*AVA*::*clr-1/RPTPΔxcd* in the text), the transmembrane and intracellular domains of the *clr-1* cDNA were amplified from *pSM*::_*p*_*rig-3*::*clr-1*, adding SalI and Acc65I sites (MVP342: AGACGTCGACGCGTATGGATATTCTGCATACT and MVP344: TATTGGTACCCCTATATGTCTTAGAGATATAG) and subcloned into *pSM*::_*p*_*rig-3*::*mCherry* using the SalI and Acc65I sites.

*pSM*::_*p*_*rig-3*::*clr-1pd*::*mCherry* (referred to as _*p*_*AVA*::*clr-1/RPTPpd* in the text) was generated using the Stratagene QuikChange site-directed mutagenesis kit to make a point mutation in the catalytic sequence of the D1 intracellular phosphatase domain of *pSM*::_*p*_*rig-3*::*clr-1*, the domain predicted to be active. The cysteine at position 1013 was replaced with a serine (C1013S). Specifically, we changed G at 3038 to C (G3038C) [18].

*pSM*::_*p*_*nlp-1*::*clr-1* (referred to as _*p*_*PHB*::*clr-1/RPTP* in the text) was generated by subcloning the *nlp-1 promoter*, flanked by the SphI and SmaI restriction sites, from *pSM*::_*p*_*nlp-1* into the SphI-SmaI fragment of *pSM*::_*p*_*rig-3*::*clr-1*.

### Fluorescence microscopy

A Zeiss Axio Imager.A1 compound fluorescent microscope and a Zeiss LSM710 confocal microscope were used to capture images of live *C*. *elegans* under 630X magnification. Worms were anesthetized on 2% agarose pads using a 2:1 ratio of 0.3 M 2,3-butanedione monoxime (BDM) and 10 mM levamisole in M9 buffer. All micrographs taken were of larval stage 4 (L4) animals, except micrographs in S2B to S2E Fig were of L2 animals.

### Phenotypic quantification

All data from micrographs were quantified using NIH ImageJ software [50], as previously described [10]. Briefly, PHB-AVA NLG-1 GRASP intensity was measured by outlining each cluster of puncta and measuring the intensity at each pixel. To account for differences in background fluorescence, background intensity was estimated by calculating the minimum intensity value in a region immediately around the puncta. This approximated value was then subtracted from the intensity for each pixel, and the sum of the adjusted values was calculated. Median intensity values were normalized to wild-type levels measured on the same day using the same settings. To measure neurite intensity, we used segmented line tool with width 10 to measure the average intensity in the anterior PHB axon and posterior AVA neurite. To account for differences in background fluorescence, background intensity was estimated by drawing a similar line in a region next to the neurite, and subtracting this value from the average intensity per pixel. PHB axon length was measured from the cell body to the distal tip of the axon. AVA axon extension was assessed by measuring the distance from the posterior end of the AVA dendrite to the anal sphincter cell.

### SDS-avoidance behavior

To test the PHB-AVA circuit function, we utilized a high-throughput SDS avoidance assay based on the previously published SDS dry drop test [4, 10]. Briefly, a day one adult hermaphrodite is placed on a dry, unseeded NGM plate. The worm is touched by a hair pick on its nose to induce backward motion by stimulating the ASH sensory neuron. Once the animal starts backing, a drop of M13 buffer or repellent (M13 buffer with 0.1% SDS) is placed on the agar behind the tail of the moving worm using a mouth pipette. The droplet placed on the agar is absorbed into the agar and the animal backs into the dry drop. The response time of the worm is the time the animal takes to stop backing into the dry drop. The response time for a minimum of 40 worms with the control M13 buffer and 40 worms to 0.1% SDS (in M13) for each genotype was recorded. The relative response index was measured by dividing the mean backing time into SDS by the mean backing time into buffer. This calculated value was then divided by the same value for wild-type animals assayed on the same day to normalize the wild-type response index to 100%. Only animals that are able to move backwards can be tested. *clr-1(e2530)* and *(n1992)* did not back sufficiently for behavioral testing.

### Statistical analysis

In the figures, the results are reported in the form of *P*-values (*P < 0.05, ** *P* < 0.01, *** *P* < 0.001, NS *P* > 0.05). *P*-values provide accurate information about whether two samples differ significantly. *P*-values are generated by a procedure that incorporates both the sample sizes and variability in samples, so that the reader is not required to multiply each standard error of the mean (obtained by assessing the length of error bars) by a factor that depends on each sample size. Error bars (standard error of the mean, or SEM) are included for the behavioral measurements throughout the manuscript. However, the NLG-1 GRASP intensity data is not normally distributed, as previously described [10], and therefore it would not be meaningful to include the SEM. Instead, we have calculated 95% confidence intervals for the medians through the bootstrap method (using the DescTools package in R [51]) for the NLG-1 GRASP data throughout the manuscript, and included them in S1 Table.

For comparing more than two NLG-1 GRASP relative median intensity values, a Kruskal-Wallis test, a nonparametric alternative to ANOVA that does not rely on a normality assumption, was first used. If a Kruskal-Wallis test yielded a *P*-value less than 0.05, or when comparing only two NLG-1 GRASP relative median intensity values, pair-wise comparisons were made using the Mann-Whitney U-test, which is a non-parametric significance test that compares medians of two independent groups. If more than one independent test was performed, *P*-values were adjusted for multiple comparisons using the Hochberg procedure. If pair-wise comparisons are not independent, the Hochberg procedure is no longer appropriate, as in Fig 5D. The Hochberg procedure is a standard method used to adjust for the tendency to incorrectly reject a null hypothesis for multiple comparisons, and can conservatively increase *P*-values.

For behavior analysis, relative SDS response indices were compared using the t-test, and *P*-values were adjusted for multiple comparisons using the Hochberg method. Statistical significance was confirmed by conducting a multi-way ANOVA model with appropriate interaction terms using the linear model procedure (R Development Core Team, 2009). For axon length, mean values were compared using an ANOVA F-test, and pair-wise comparisons were made using the t-test, and adjusted for multiple comparisons using the Hochberg method. Microsoft Excel and R statistical computing software [51] were used for all statistical tests.

## Supporting information

S1 Fig PHB axon length is slightly reduced, and AVA dendrites are slightly longer in *clr-1/RPTP* mutants. (A) Quantification of PHB axon length in wild-type, *clr-1/RPTP(e1745)*, and *ced-10/Rac1(n1993)* animals (n>40). ***P<0.001, t-test, comparison with wild-type. *P*-values were adjusted for multiple comparisons using the Hochberg method. (B) Quantification of a reduction in NLG-1 GRASP fluorescence in *clr-1/RPTP(e1745)* animals and no significant difference in NLG-1 GRASP fluorescence in *ced-10/Rac1(n1993)* animals, indicating that a reduction in length is not sufficient to cause a reduction in PHB-AVA synapses (n≥40). ***P<0.001, NS, not significant, U-test, comparison with wild-type. *P*-values were adjusted for multiple comparisons using the Hochberg method. 95% confidence intervals for the medians are included in S1 Table. (C) Schematic of the region measured in D to assess AVA neurite extension: the distance between the end of the AVA neurite and the posterior tip of the gut. Note that the shorter this distance is, the longer the extension of the AVA neurites. (D) Quantification of the relative distance from the AVA neurite to the posterior tip of the gut in wild-type and *clr-1/RPTP(e1745)* mutants. AVA was labeled with mCherry (_*p*_*AVA*::*mCherry*). Note that the shorter distance in *clr-1/RPTP* mutants indicates slightly increased AVA neurite length.
(TIF)

S2 Fig CLR-1/RPTP is not required for trafficking of presynaptic RAB-3, ELKS-1 or postsynaptic NLG-1 to the PHB-AVA synaptic region. (A) Quantification of similar median neurite fluorescence intensity in wild-type and *clr-1/RPTP* animals using the same promoters that drive the PHB-AVA NLG-1 GRASP marker in PHB and AVA neurons. NS, not significant, U-test, comparison with wild-type. *P*-values were adjusted for multiple comparisons using the Hochberg method. (B to D) Representative schematics and micrographs of wild-type (B and C) and *clr-1/RPTP(e1745)* (D and E) labeled with the presynaptic vesicle marker *mCherry*::*rab-3* expressed in PHB (_*p*_*PHB*::*mCherry*::*rab-3)*. (E to H) Representative schematics and micrographs of wild-type (F and G) and *clr-1/RPTP(e1745)* (H and I) labeled with the presynaptic active zone marker *GFP*::*elks-1* expressed in PHB (_*p*_*PHB*:::*GFP*::*elks-1*). Representative schematics and micrographs of wild-type (J and K) and *clr-1/RPTP(e1745)* (L and M) labeled with the postsynaptic marker *nlg-1*::*YFP* expressed in AVA (_*p*_*AVA*::*nlg-1*::*YFP*). (N) Quantification of no significant difference in NLG-1 GRASP fluorescence in the region of the preanal ganglion in _*p*_*PHB*::*mCherry*::*rab-3*, _*p*_*PHB*:::*GFP*::*elks-1*, or _*p*_*AVA*::*nlg-1*::*YFP* in *clr-1/RPTP(e1745)* mutants compared with wild-type animals (n≥24). NS, not significant, U-test. *P*-values were adjusted for multiple comparisons using the Hochberg method.
(TIF)

S3 Fig CLR-1/RPTP is expressed in AVA and not PHB neurons. (A) Wild-type animals expressing GFP under the direction of the *clr-1* promoter and mCherry under the direction of the *rig-3* promoter, which drives expression in AVA neurons and a few other cells in the head [53]. The _*p*_*clr-1*::*GFP* transcriptional fusion was expressed in AVA neurons (arrows), which were identified based on expression of _*p*_*rig-3*::*mCherry*, their cell body position and axon morphology. (B) Wild-type animals expressing _*p*_*clr-1*::*GFP* and mCherry under the direction of a promoter that, in the posterior of the worm, is specifically expressed in PHB neurons (_*p*_*nlp-1*::*mCherry*). The _*p*_*clr-1*::*GFP* transcriptional fusion was not expressed in PHB neurons (arrow indicates placement of PHB neurons).
(TIF)

S4 Fig *clr-1/RPTP* mutants display defective synaptic partner recognition between AVA and VA10 neurons. (A) Representative diagrams and micrographs of NLG-1 GRASP fluorescence labeling synapses between AVA and VA and DA motorneurons in wild-type and *clr-1(e1745)* mutants. (B) Quantification of a severe reduction in median NLG-1 GRASP fluorescence in *clr-1(e1745)* animals in comparison to wild-type animals in the region between the VA10 and DA7 neurons, where synapses between AVA and VA10 are observed in wild-type animals. ***P<0.001, U-test, comparison with wild type.
(TIF)

S1 Table 95% confidence intervals for NLG-1 GRASP synaptic intensity data. (DOCX)

S2 Table Relative median NLG-1 GRASP intensity and relative mean SDS response index for individual transgenic lines. (DOCX)

S3 Table Data underlying graphs. (XLSX)
