# Supplementary figures and images for: The receptor protein tyrosine phosphatase CLR-1 is required for synaptic partner recognition
Source: PLoS Genet. 2018 May 9;14(5):e1007312. doi: 10.1371/journal.pgen.1007312 (PMC5942785; doi:10.1371/journal.pgen.1007312)

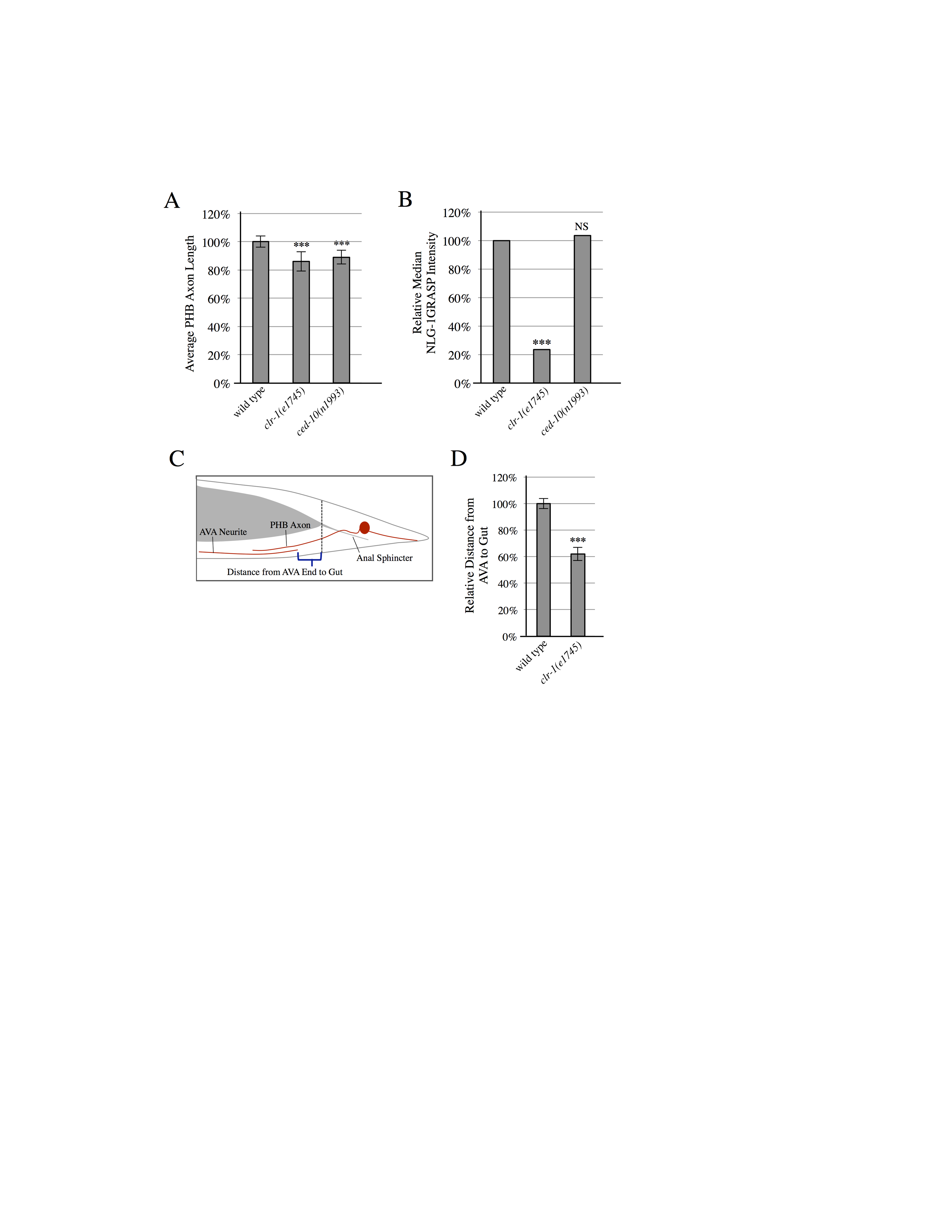

Supplement: S1 Fig — (A) Quantification of PHB axon length in wild-type, clr-1/RPTP(e1745), and ced-10/Rac1(n1993) animals (n>40). ***P<0.001, t-test, comparison with wild-type. P-values were adjusted for multiple comparisons using the Hochberg method. (B) Quantification of a reduction in NLG-1 GRASP fluorescence in clr-1/RPTP(e1745) animals and no significant difference in NLG-1 GRASP fluorescence in ced-10/Rac1(n1993) animals, indicating that a reduction in length is not sufficient to cause a reduction in PHB-AVA synapses (n≥40). ***P<0.001, NS, not significant, U-test, comparison with wild-type. P-values were adjusted for multiple comparisons using the Hochberg method. 95% confidence intervals for the medians are included in S1 Table. (C) Schematic of the region measured in D to assess AVA neurite extension: the distance between the end of the AVA neurite and the posterior tip of the gut. Note that the shorter this distance is, the longer the extension of the AVA neurites. (D) Quantification of the relative distance from the AVA neurite to the posterior tip of the gut in wild-type and clr-1/RPTP(e1745) mutants. AVA was labeled with mCherry (pAVA::mCherry). Note that the shorter distance in clr-1/RPTP mutants indicates slightly increased AVA neurite length. (TIF) [file pgen.1007312.s001.tif]

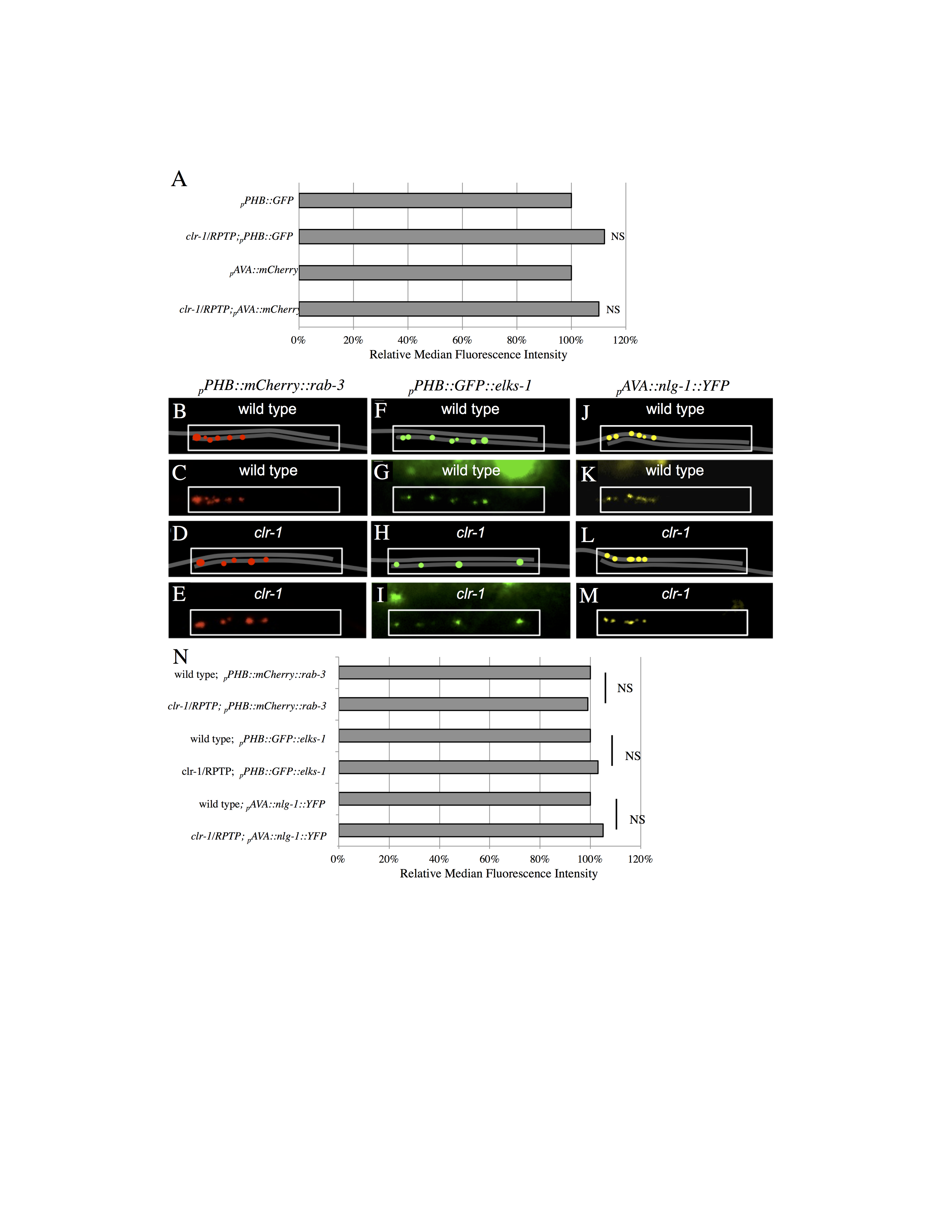

Supplement: S2 Fig — (A) Quantification of similar median neurite fluorescence intensity in wild-type and clr-1/RPTP animals using the same promoters that drive the PHB-AVA NLG-1 GRASP marker in PHB and AVA neurons. NS, not significant, U-test, comparison with wild-type. P-values were adjusted for multiple comparisons using the Hochberg method. (B to D) Representative schematics and micrographs of wild-type (B and C) and clr-1/RPTP(e1745) (D and E) labeled with the presynaptic vesicle marker mCherry::rab-3 expressed in PHB (pPHB::mCherry::rab-3). (E to H) Representative schematics and micrographs of wild-type (F and G) and clr-1/RPTP(e1745) (H and I) labeled with the presynaptic active zone marker GFP::elks-1 expressed in PHB (pPHB:::GFP::elks-1). Representative schematics and micrographs of wild-type (J and K) and clr-1/RPTP(e1745) (L and M) labeled with the postsynaptic marker nlg-1::YFP expressed in AVA (pAVA::nlg-1::YFP). (N) Quantification of no significant difference in NLG-1 GRASP fluorescence in the region of the preanal ganglion in pPHB::mCherry::rab-3, pPHB:::GFP::elks-1, or pAVA::nlg-1::YFP in clr-1/RPTP(e1745) mutants compared with wild-type animals (n≥24). NS, not significant, U-test. P-values were adjusted for multiple comparisons using the Hochberg method. (TIF) [file pgen.1007312.s002.tif]

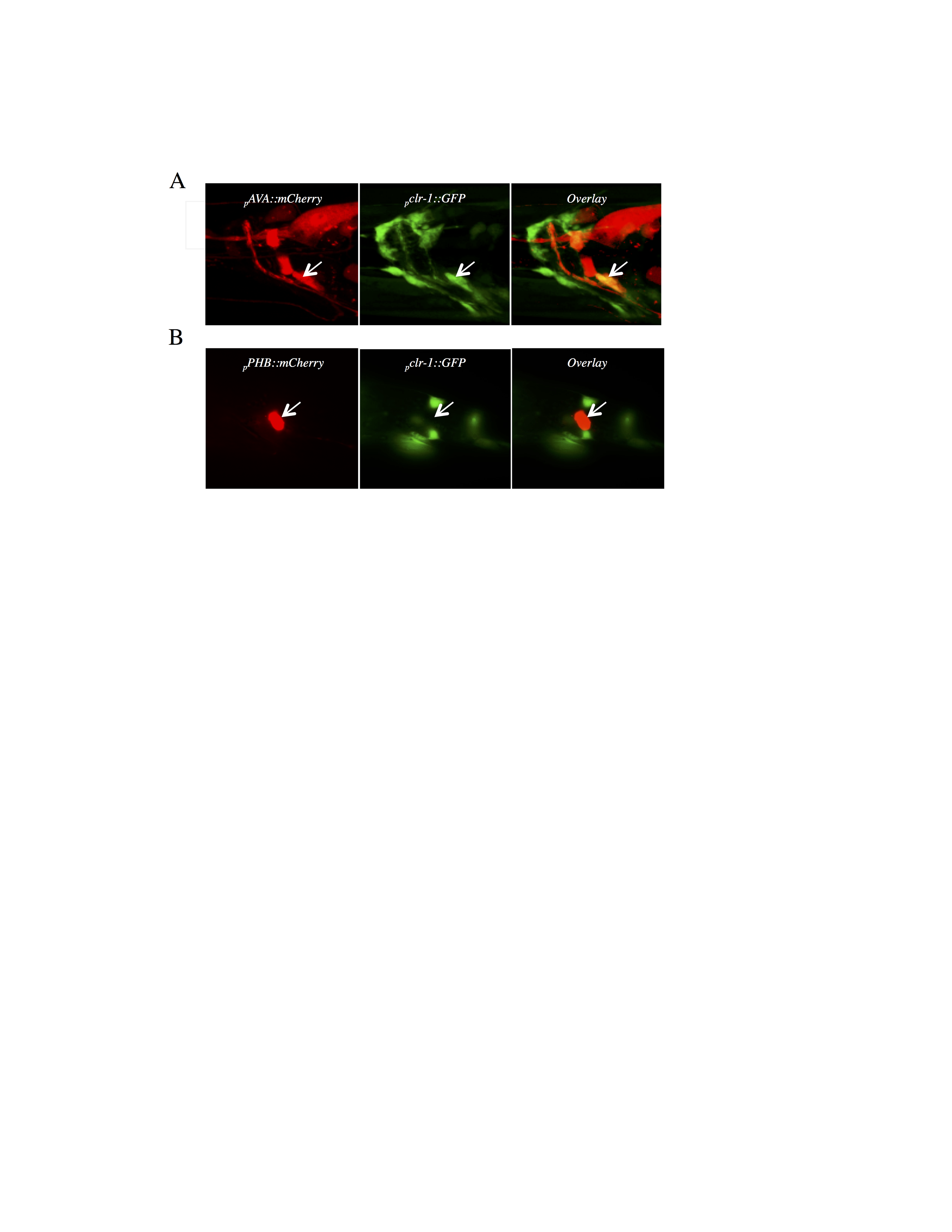

Supplement: S3 Fig — (A) Wild-type animals expressing GFP under the direction of the clr-1 promoter and mCherry under the direction of the rig-3 promoter, which drives expression in AVA neurons and a few other cells in the head [53]. The pclr-1::GFP transcriptional fusion was expressed in AVA neurons (arrows), which were identified based on expression of prig-3::mCherry, their cell body position and axon morphology. (B) Wild-type animals expressing pclr-1::GFP and mCherry under the direction of a promoter that, in the posterior of the worm, is specifically expressed in PHB neurons (pnlp-1::mCherry). The pclr-1::GFP transcriptional fusion was not expressed in PHB neurons (arrow indicates placement of PHB neurons). (TIF) [file pgen.1007312.s003.tif]

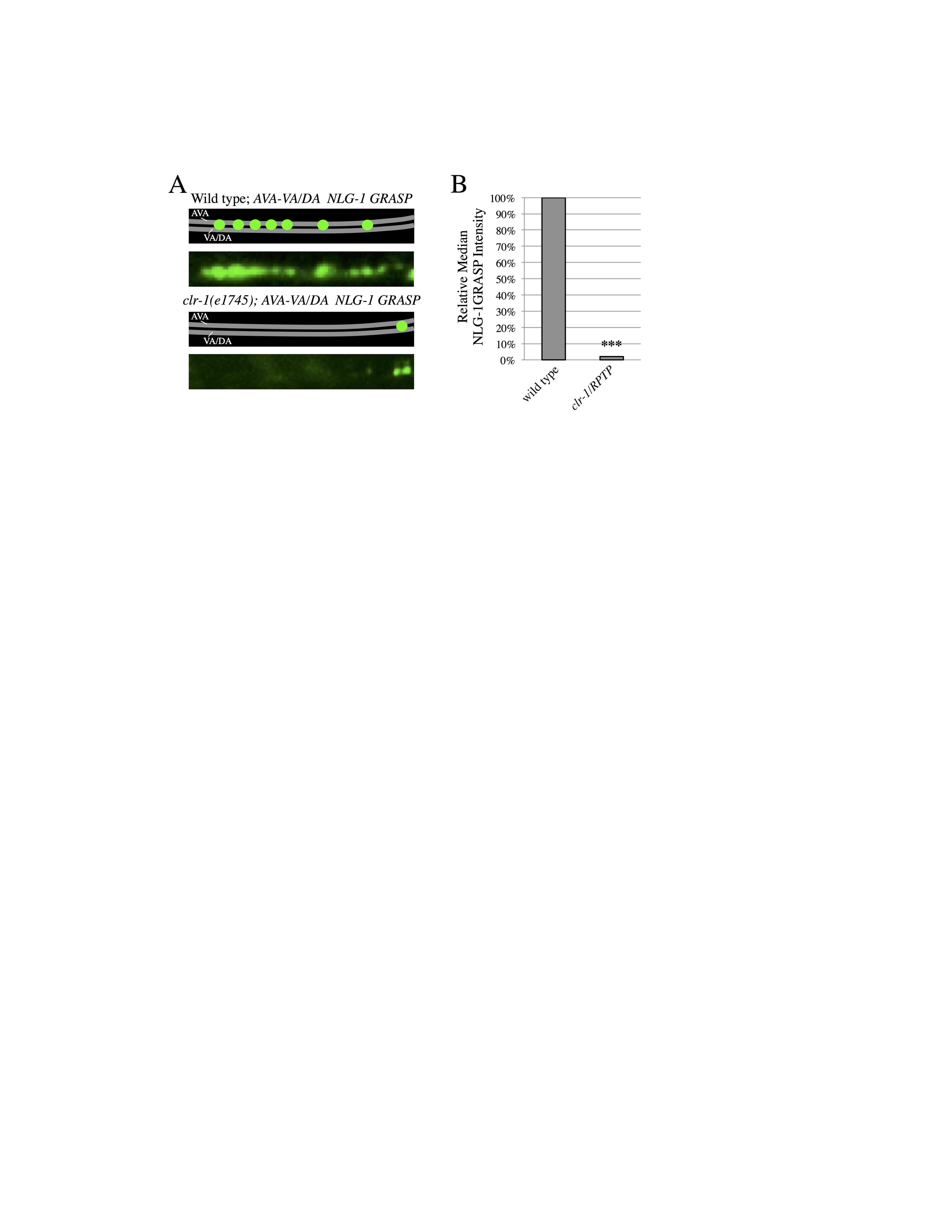

Supplement: S4 Fig — (A) Representative diagrams and micrographs of NLG-1 GRASP fluorescence labeling synapses between AVA and VA and DA motorneurons in wild-type and clr-1(e1745) mutants. (B) Quantification of a severe reduction in median NLG-1 GRASP fluorescence in clr-1(e1745) animals in comparison to wild-type animals in the region between the VA10 and DA7 neurons, where synapses between AVA and VA10 are observed in wild-type animals. ***P<0.001, U-test, comparison with wild type. (TIF) [file pgen.1007312.s004.tif]
