# Supplementary material for: The receptor protein tyrosine phosphatase CLR-1 is required for synaptic partner recognition
Source: PLoS Genet. 2018 May 9;14(5):e1007312. doi: 10.1371/journal.pgen.1007312 (PMC5942785; doi:10.1371/journal.pgen.1007312)
Supplement: S1 Table — (DOCX) [file pgen.1007312.s005.docx]

**Supporting Table 1. 95% Confidence Intervals for NLG-1 GRASP synaptic intensity data**

| Figure Number | Figure Letter | Genotype | Array | 95% Confidence Interval |
| --- | --- | --- | --- | --- |
|  |  |  |  |  |
| 1 | H | wild type |  | 82% - 116% |
| 1 | H | *clr-1(e1745)/RPTP* |  | 19% - 29% |
| 1 | H | *clr-1(e2530)/RPTP* |  | 32% - 56% |
| 1 | H | *clr-1(n1992)/RPTP* |  | 50% - 91% |
| 2 | B | wild type |  | 93% - 118% |
| 2 | B | *clr-1/RPTP* | *_p_AVA::clr-1/RPTP* | 72% - 111% |
| 2 | B | *clr-1/RPTP* | *_p_PHB::clr-1/RPTP* | 12% - 22% |
| 2 | B | *clr-1/RPTP* | *_p_AVA::clr-1/RPTPΔxcd* | 46% - 63% |
| 2 | B | *clr-1/RPTP* | *_p_AVA::clr-1/RPTPpd* | 22% - 49% |
| 3 | C | wild type 20°C |  | 68% - 130% |
| 3 | C | *clr-1/RPTP* 20°C |  | 7% - 29% |
| 3 | C | wild type 16°C |  | 43% - 126% |
| 3 | C | *clr-1/RPTP* 16°C |  | 39% - 66% |
| 4 | B | wild type |  | 81% - 113% |
| 4 | B | wild type | *_p_AVA::clr-1/RPTP* | 131% - 185% |
| 5 | B | wild type |  | 79% - 109% |
| 5 | B | *unc-40/DCC* |  | 24% - 53% |
| 5 | B | *unc-6/Netrin* |  | 38% - 76% |
| 5 | B | *clr-1;unc-40* |  | 13% - 34% |
| 5 | B | *clr-1;unc-6* |  | 27% - 53% |
| 5 | B | wild type | *_p_AVA::unc-6/Netrin* | 120% - 162% |
| 5 | B | *clr-1/RPTP* | *_p_AVA::unc-6/Netrin* | 24% - 40% |
| 5 | D | wild type |  | 82% - 111% |
| 5 | D | *clr-1/+* |  | 56% - 124% |
| 5 | D | *unc-40/+* |  | 67% - 96% |
| 5 | D | *unc-6/+* |  | 89% - 137% |
| 5 | D | *clr-1/+; unc-40/+* |  | 39% - 60% |
| 5 | D | *clr-1/+; unc-6/+* |  | 64% - 83% |
| S 1 | B | wild type |  | 76% - 121% |
| S 1 | B | *ced-10(n1993)* |  | 91% - 133% |

Please note that if the data were normally distributed, then a 95% confidence interval for the median would be expected to be about four times as wide as the SEM.
