# Supplementary material for: The receptor protein tyrosine phosphatase CLR-1 is required for synaptic partner recognition
Source: PLoS Genet. 2018 May 9;14(5):e1007312. doi: 10.1371/journal.pgen.1007312 (PMC5942785; doi:10.1371/journal.pgen.1007312)
Supplement: S2 Table — (DOCX) [file pgen.1007312.s006.docx]

**Supporting Table 2. Relative Median NLG-1 GRASP Intensity and Relative Mean SDS Response Index for individual transgenic lines.**

| Figure Number | Figure Letter | Genotype | Transgene | Relative Median NLG-1 GRASP Intensity | Relative Mean SDS Response Index | Non-Adjusted p-value, comparison with wild type |
| --- | --- | --- | --- | --- | --- | --- |
| 2 | B | *clr-1/RPTP* | *iyEx97; _p_AVA::clr-1/RPTP* | 82% |  | 9.14E-2 |
| 2 | C | *clr-1/RPTP* | *iyEx97; _p_AVA::clr-1/RPTP* |  | 99% | 9.0E-01 |
| 2 | B | *clr-1/RPTP* | *iyEx101; _p_AVA::clr-1/RPTP* | 112% |  | 5.8E-01 |
| 2 | C | *clr-1/RPTP* | *iyEx101; _p_AVA::clr-1/RPTP* |  | 94% | 5.4E-01 |
| 2 | B | *clr-1/RPTP* | *iyEx364; _p_PHB::clr-1/RPTP* | 10% |  | 2.2E-16 |
| 2 | C | *clr-1/RPTP* | *iyEx364; _p_PHB::clr-1/RPTP* |  | 235% | 2.7E-06 |
| 2 | B | *clr-1/RPTP* | *iyEx365; _p_PHB::clr-1/RPTP* | 16% |  | 5.6E-12 |
| 2 | C | *clr-1/RPTP* | *iyEx365; _p_PHB::clr-1/RPTP* |  | 207% | 4.1E-14 |
| 2 | B | *clr-1/RPTP* | *iyEx366, _p_PHB::clr-1/RPTP* | 22% |  | 6.8E-11 |
| 2 | C | *clr-1/RPTP* | *iyEx366; _p_PHB::clr-1/RPTP* |  | 261% | 1.1E-22 |
| 2 | B | *clr-1/RPTP* | *iyEx133; _p_AVA::clr-1/RPTPΔxcd* | 67% |  | 3.4E-02 |
| 2 | C | *clr-1/RPTP* | *iyEx133; _p_AVA::clr-1/RPTPΔxcd* |  | 229% | 2.7E-08 |
| 2 | B | *clr-1/RPTP* | *iyEx134; _p_AVA::clr-1/RPTPΔxcd* | 48% |  | 2.0E-04 |
| 2 | C | *clr-1/RPTP* | *iyEx134; _p_AVA::clr-1/RPTPΔxcd* |  | 156% | 2.2E-05 |
| 2 | B | *clr-1/RPTP* | *iyEx135; _p_AVA::clr-1/RPTPΔxcd* | 33% |  | 6.6E-05 |
| 2 | C | *clr-1/RPTP* | *iyEx135; _p_AVA::clr-1/RPTPΔxcd* |  | 218% | 8.6E-09 |
| 2 | B | *clr-1/RPTP* | *iyEx362; _p_AVA::clr-1/RPTPΔxcd* | 38% |  | 3.2E-04 |
| 2 | C | *clr-1/RPTP* | *iyEx362; _p_AVA::clr-1/RPTPΔxcd* |  | 222% | 2.1E-12 |
| 2 | B | *clr-1/RPTP* | *iyEx169; _p_AVA::clr-1/RPTPpd* | 60% |  | 3.3E-03 |
| 2 | C | *clr-1/RPTP* | *iyEx169; _p_AVA::clr-1/RPTPpd* |  | 255% | 8.7E-13 |
| 2 | B | *clr-1/RPTP* | *iyEx170; _p_AVA::clr-1/RPTPpd* | 42% |  | 4.2E-05 |
| 2 | C | *clr-1/RPTP* | *iyEx170; _p_AVA::clr-1/RPTPpd* |  | 190% | 9.0E-09 |
| 2 | B | *clr-1/RPTP* | *iyEx174; _p_AVA::clr-1/RPTPpd* | 26% |  | 2.4E-05 |
| 2 | C | *clr-1/RPTP* | *iyEx174; _p_AVA::clr-1/RPTPpd* |  | 218% | 1.2E-08 |
| 4 | B | *Wild type* | *iyEx97; _p_AVA::clr-1/RPTP* | 157% |  | 1.4E-03 |
| 4 | C | *Wild type* | *iyEx97; _p_AVA::clr-1/RPTP* |  | 82% | 3.5E-02 |
| 4 | B | *Wild type* | *iyEx101; _p_AVA::clr-1/RPTP* | 170% |  | 7.6E-06 |
| 4 | C | *Wild type* | *iyEx101; _p_AVA::clr-1/RPTP* |  | 90% | 3.0E-01 |
